# Causal relationship between circulating immune cells and the risk of type 2 diabetes: a Mendelian randomization study

**DOI:** 10.3389/fendo.2023.1210415

**Published:** 2023-05-25

**Authors:** Jin Li, Qingmin Niu, Aiwei Wu, Yuchu Zhang, Liquan Hong, Hu Wang

**Affiliations:** Key Laboratory of Aging and Cancer Biology of Zhejiang Province, School of Basic Medical Sciences, The Third People’s Hospital of Deqing, Deqing Hospital of Hangzhou Normal University, Hangzhou Normal University, Hangzhou, China

**Keywords:** circulating immune cells, type 2 diabetes (T2D), Mendelian randomization (MR), genome wide association study (GWAS), monocyte

## Abstract

**Objectives:**

Though type 2 diabetes (T2D) has been known as a metabolic disease caused by multiple factors, the etiology remains insufficiently understood. Here, we aimed to figure out whether circulating immune cell profiles causally impact T2D liability.

**Methods:**

We applied one genome-wide association study (GWAS) summary statistics of blood traits in 563,085 participants from the Blood Cell Consortium and another GWAS of flow cytometric profile of lymphocyte subsets comprising 3,757 Sardinians to identify genetically predicted blood immune cells. We also obtained GWAS summary statistics in 898,130 individuals from the DIAGRAM Consortium to evaluate genetically predicted T2D. We primarily used inverse variance weighted (IVW) and weighted median methods to perform Mendelian randomization analyses and sensitivity analyses to evaluate heterogeneity and pleiotropy.

**Results:**

For circulating blood leukocyte and its subpopulations, the increase of genetically predicted circulating monocyte count was causally correlated with a higher risk of T2D [odds ratio (OR) = 1.06, 95% confidence interval (CI) = 1.02–1.10, p = 0.0048]. For lymphocyte subsets, CD8^+^ T cell and CD4^+^ CD8^dim^ T cell count were identified with causal effect on T2D susceptibility (CD8^+^ T cell: OR = 1.09, 95% CI = 1.03–1.17, p = 0.0053; CD4^+^ CD8^dim^ T cell: OR = 1.04, 95% CI = 1.01–1.08, p = 0.0070). No pleiotropy was determined.

**Conclusions:**

These findings demonstrated that higher circulating monocyte and T-lymphocyte subpopulation predicted increased T2D susceptibility, which confirmed the immunity predisposition for T2D. Our results may have the potential to provide new therapeutic targets for the diagnosis and treatment of T2D.

## 1 Introduction

Type 2 diabetes (T2D) is a major cause of disability and death globally, with approximately half of these deaths occurring before the age of 70 (WHO). T2D and its complications continue to pose a growing burden worldwide (1). While T2D was traditionally considered a metabolic disease resulting from insulin resistance and insufficient insulin production, recent genetic, epidemiological, and cytokine profiling studies have supported the idea that chronic low-grade activation of the immune system may also play a role in the development of T2D (2–7). Inflammatory immune cells can infiltrate adipose tissue and release inflammatory cytokines, contributing to systemic inflammation and worsening T2D (8). Therefore, T2D has been suggested to be categorized into severe insulin-deficient diabetes (SIDD), severe insulin-resistant diabetes (SIRD), mild obesity-related diabetes (MOD), and mild age-related diabetes (MARD) (9). Higher leukocyte count, including monocyte and CD4^+^ T cell count, has been found in SIRD and MOD patients compared to SIDD and MARD patients, suggesting that specific dysregulation of immune cells may closely relate to insulin resistance in these groups (10). However, much of the evidence to date is based on observational studies that may be limited by confounding factors and reverse causality. Few studies have evaluated the causal relationship between circulating immune cell count and T2D onset.

Mendelian randomization (MR) was initially developed to be an alternative option for randomized controlled trials (RCTs), providing reliable causal evidence between exposures and outcomes through genetic variants (11). Since genetic variants are randomly assigned at conception prior to disease onset, MR has been an efficient tool to identify the causal relationship independent of confounding factors and avoid reverse causality (12). Several previous MR analyses have identified that risk factors such as percentage of body fat, systolic blood pressure, and iron status may predict T2D susceptibility (13, 14). In the present study, we determined the pathophysiological role of the immune system in the development of T2D, specifically the subtypes of leukocyte, using the recent large-scale genome-wide association study (GWAS) summary statistics on blood traits (15) and another GWAS including specific immune cell profiling of European ancestry by flow cytometry (16). Our aim was to determine whether there is a causal link between circulating immune cells and T2D incidence. Understanding the risk factors associated with T2D progression will aid in the development of novel therapies to address this growing public health concern.

## 2 Materials and methods

### 2.1 Study design

**Figure 1:**
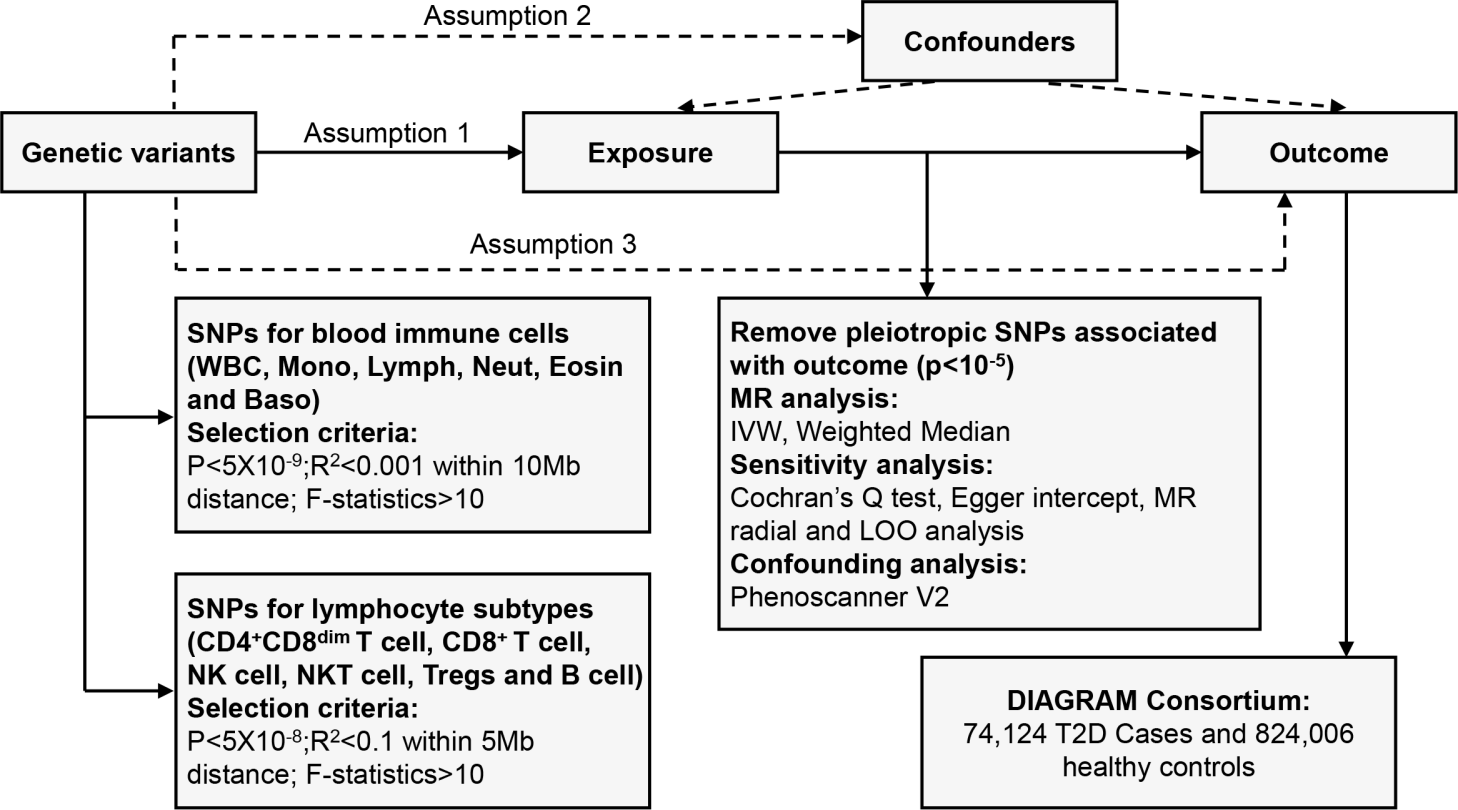
Overview of the overall MR design. Assumption 1, instrument variables are robustly related to exposure; Assumption 2, instrument variables are not related to confounders; Assumption 3, instrument variables are related to outcome only through exposure. SNPs, single-nucleotide polymorphisms; LD, linkage disequilibrium; IVW, inverse variance weighted; LOO, leave-one-out; MR, Mendelian randomization.

We systematically determined the causal effects of peripheral blood immune cells or lymphocyte subtypes on the risk of T2D using a two-sample MR study. There are three fundamental assumptions that should be followed in the MR study (17). First, the genetic variants should be closely associated with exposure. Second, they should be independent of potential confounders. Last but not least, these genetic instruments should affect the outcome only by exposure. The overall design is shown in **Figure 1**.

### 2.2 Data sources for exposure and outcome

For the exposure instrument, we used the summary statistics from the recent large-scale GWAS on blood cell traits from Blood Cell Consortium (BCX), which includes 563,085 European ancestry participants (15). Genetic variants associated with the level of circulating white blood cell, lymphocyte, monocyte, neutrophil, eosinophil, and basophil count were obtained from this GWAS. Flow cytometric profile of lymphocyte subsets including HLA DR^+^ natural killer (NK) cells, NKT cells, CD4 regulatory T cells (Tregs), CD4^+^ CD8^dim^ T cells, CD8^+^ T cells, and B cells were derived from the recent GWAS summary statistics including 3,757 Sardinians (16). Outcome data for T2D were taken from large-scale case–control GWAS statistics from the DIAGRAM Consortium involving 74,124 T2D cases and 824,006 controls of European ancestry (18). The average age of individuals in this study was 55 years, with 51.8% being male. The Haplotype Reference Consortium (HRC) reference panel was applied during imputation. The data are publicly available at the website: http://diagram-consortium.org. Detailed information is shown in **Supplementary Table 1**.

### 2.3 Instrument selection

Considering the large number of single-nucleotide polymorphisms (SNPs) achieving genome-wide significance (p < 5 × 10^−8^) for the blood cell traits from BCX, we used a more stringent association threshold (p < 5 × 10^−9^) to select genetic instrumental variables (IVs) (19). To obtain independent IVs, we performed clumping (R^2^ < 0.001 within a 1,000-kb distance) based on the linkage disequilibrium (LD) reference panel of the 1000 Genomes Project. Referring to the relatively modest-scale GWAS regarding lymphocyte subpopulations, we applied a p-value cutoff of 5 × 10^−8^ and a more relaxed clumping threshold (R^2^ < 0.1 within a 500-kb distance) (20). Additionally, to avoid bias brought by weak instruments, we considered IVs with F statistics >10 as strong instruments and reserved them for the following analysis. We then extracted IVs from outcome T2D summary statistics and removed those harboring potential pleiotropic effects on T2D (p < 10^−5^) as previously reported (21). We harmonized the exposure and outcome SNPs to ensure that effect estimates were aligned for the same effect allele. Palindromic SNPs with intermediate effect allele frequencies (EAFs > 0.42) or SNPs with incompatible alleles were discarded (20).

### 2.4 Statistical analysis

As our primary analysis, we used the random-effects inverse variance weighted (IVW) and weighted median (WM) methods, providing an estimate of the effect of the exposure on the outcome when MR assumptions are valid. For a single genetic variant identified, we performed a Wald ratio estimate in our MR studies. As a secondary sensitivity analysis, we applied Cochran’s Q test (p < 0.05) to estimate residual heterogeneity for the IVW model and the MR-Egger intercept test (p < 0.05) to indicate potential pleiotropy on causal estimates. We also performed a radial MR test to identify pleiotropic outliers (22). We presented SNPs that contribute robustly to heterogeneity. We then removed these outliers and reanalyzed these data. Finally, we made a leave-one-out (LOO) analysis to find out whether an SNP could drive the bias of causal estimate. Previous MR analyses have confirmed 34 risk factors (body fat percentage, systolic blood pressure, birthweight, total and HDL cholesterol, 25-hydroxyvitamin D, resting heart rate, age at menarche, etc.) are causally related to T2D (13). We searched the Phenoscanner V2 website (http://www.phenoscanner.medschl.cam.ac.uk/) to find out SNPs showing suggestive association (p < 10^−5^) with those risk factors. Then, we repeated the IVW analysis after removing these SNPs. Since there are multiple tests in this study, we adopted a Bonferroni-corrected significant threshold of 0.05/6. Analyses were implemented by the package TwoSampleMR (version 0.5.6) and RadialMR (version 1.0) in R (version 3.4).

## 3 Results

### 3.1 Causal estimates between peripheral immune cells and T2D

After the selection and harmonization of IVs, we utilized 361 SNPs for monocyte cell count, 327 SNPs for white blood cell (WBC) count, 334 SNPs for lymphocyte cell count, 247 SNPs for neutrophil cell count, 296 SNPs for eosinophil cell count, and 104 SNPs for basophil cell count for MR analysis. All SNPs had F statistics above 10, demonstrating their suitability as strong instruments. The harmonized data are presented in **Supplementary Tables 2–7**.

**Figure 2:**
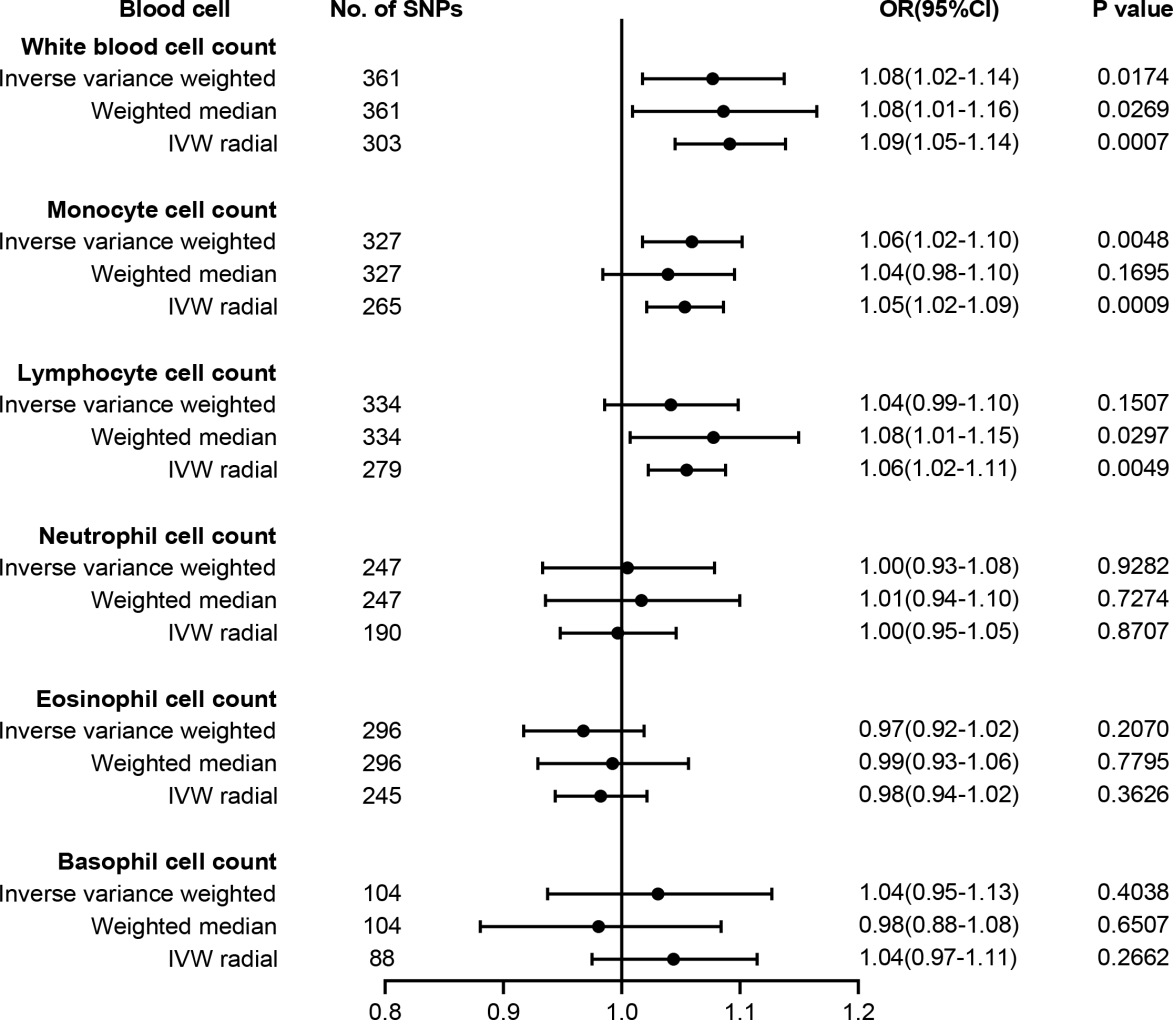
Forest plot for the causal effect of circulating immune cells on the risk of T2D derived from inverse variance weighted (IVW), weighted median, and IVW radial. OR, odds ratio; CI, confidence interval; T2D, type 2 diabetes.

The causal effect estimates of peripheral immune cell count on T2D susceptibility are summarized in **Figure 2**. Notably, our results demonstrated a strong causal association between a higher liability of T2D and an increased level of circulating monocyte cell count using IVW analysis [odds ratio (OR) = 1.06, 95% confidence interval (CI) = 1.02–1.10, p = 0.0048], which remained significant even after Bonferroni correction. Similar positive trends with weighted median (OR = 1.04, 95% CI = 0.98–1.10, p = 0.1695) methods were observed. WBC count might have a positive causal relationship with the risk of T2D using either IVW (OR = 1.08, 95% CI = 1.02–1.14, p = 0.0174) or WM (OR = 1.08, 95% CI = 1.01–1.16, p = 0.0269) methods. However, no obvious association was observed between lymphocyte, neutrophil, eosinophil, or basophil cell count and T2D susceptibility.

The results of sensitivity analyses are summarized in **Supplementary Table 8**. Although heterogeneity was observed with a Cochran’s Q-derived p-value <0.05, the causal estimate was acceptable when using the random-effects IVW method (23). The p-value for the MR-Egger intercept was above 0.05, indicating that no obvious pleiotropy was found (**Supplementary Table 8**). Radial MR identified some pleiotropic outliers, and similar causal estimates for T2D risk were obtained following the outliers’ removal (**Figure 2**). LOO analysis further revealed that no SNP introduced pleiotropic bias in our causal estimates (**Supplementary Figure 1**).

A recent summary-level MR study verified 34 causal risk factors for T2D, including 25-hydroxyvitamin D, age at menarche systolic BP, resting heart rate, body fat percentage, birthweight, HDL, and total cholesterol (13). To exclude potential SNPs associated with these traits, we searched the Phenoscanner manually and finally found 25 SNPs for monocyte and 28 SNPs for WBC possibly associated with these risk factors (**Supplementary Table 9**). After we removed these SNPs, the causality remained significant in monocyte cell count (OR = 1.06, 95% CI = 1.01–1.10, p = 0.0094) and WBC count (OR = 1.07, 95% CI = 1.01–1.13, p = 0.0166).

### 3.2 Causal estimates between circulating lymphocyte subtypes and T2D

**Figure 3:**
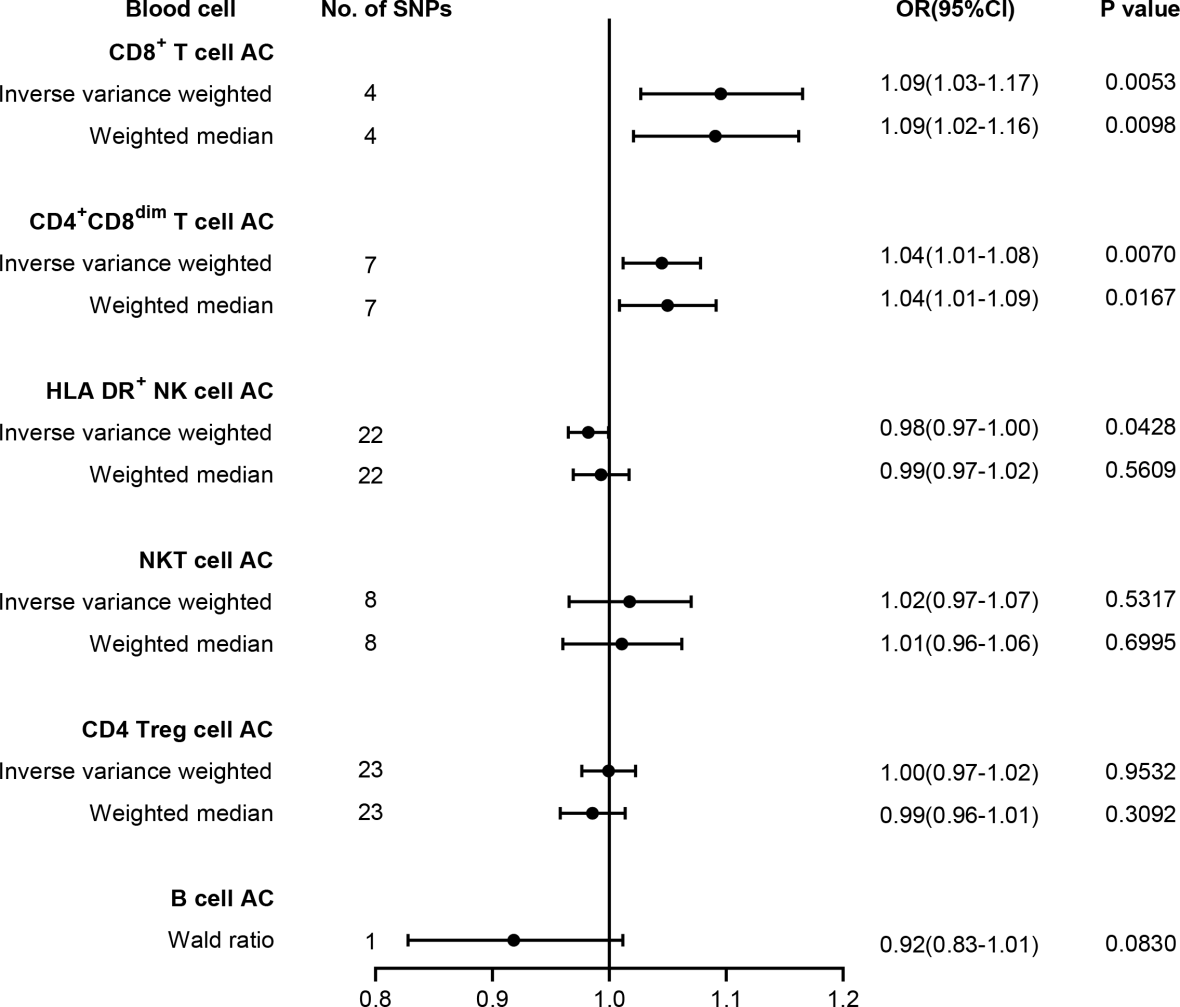
Forest plot for the causal effect of lymphocyte subtypes on the risk of T2D derived from inverse variance weighted (IVW), weighted median, and Wald ratio. AC, absolute count; OR, odds ratio; CI, confidence interval; T2D, type 2 diabetes.

To test whether lymphocyte subtypes are causally associated with T2D risk, we further carried out MR analysis between T2D and absolute count of lymphocyte subpopulations, comprising HLA DR^+^ NK cells, NKT cells, CD4 Tregs, CD4^+^ CD8^dim^ T cells, CD8^+^ T cells, and B cells. Our results are summarized in **Figure 3**, and the harmonized data are presented in **Supplementary Table 10**. Briefly, genetic liability to higher levels of CD8^+^ T cell count (OR = 1.09, 95% CI = 1.03–1.17, p = 0.0053) and CD4^+^ CD8^dim^ T cell count (OR = 1.04, 95% CI = 1.01–1.08, p = 0.0070) predicted a higher risk of T2D, while genetic liability to lower levels of HLA DR^+^ NK cell count (OR = 0.98, 95% CI = 0.97–1.00, p = 0.0428) may predict a higher risk of T2D. However, no significant association was found between T2D risk and the absolute count of other cell subtypes, such as NKT cells, CD4 Tregs, or B cells. No pleiotropy and heterogeneity were identified in the MR-Egger intercept test and Cochran’s Q test (**Supplementary Table 8**). LOO analysis revealed that none of the SNPs could individually affect the causal estimate (**Supplementary Figure 2**).

## 4 Discussion

The MR analysis has been commonly applied to elucidate the potential causal relationship between risk factors and diseases. In this study, we provide evidence to present the inverse causal association between monocyte count and T2D from a genetic perspective using MR analysis. This is in line with previous results from the INTERCEPT-T2D EU-supported project (10). However, there are also reports that the number and distribution of peripheral blood monocytes, including classical, non-classical, and intermediate types, do not differ between diabetic patients and healthy individuals, except for a significant change in the ratio of pro-inflammatory M1 to anti-inflammatory M2 (24). These controversial results may relate to methodological flaws, such as controlling for confounding factors. The MR analysis method we used here can obtain clear causal relationships between peripheral blood monocyte count and T2D risk by controlling residual confounding factors and reverse causality analysis. We applied several sensitivity tests to evaluate the robustness of the results and minimize potential bias derived from pleiotropic effects. In recent years, this method has identified various T2D risk factors with clear causal relationships, including blood iron level, percent of body fat, and systolic BP (13, 14). Furthermore, we found a weak positive causal relationship between total peripheral blood leukocyte count and T2D liability, which is consistent with the increase in leukocyte count in T2D patients (6, 10).

It has been known that the pathological characteristics of T2D are due to impaired insulin secretion and sensitivity caused by oxidative stress, endoplasmic reticulum stress, and ectopic lipid deposition in the muscle, liver, and pancreas (25). All these dysfunctions may recruit inflammatory macrophages from circulating monocytes, whereas these infiltrated inflammatory macrophages may release plenty of inflammatory factors, causing chronic low-grade inflammation and finally exacerbating the dysfunction of these tissues. It is worth noting that, with the rapid development of high-throughput single-cell transcriptomics technology, we have learned that there seemed to be several subpopulations of peripheral tissue-resident macrophages with diverse functions except for pro-inflammatory effects. Lyve1^+^ macrophage populations can control adipose tissue metabolism plasticity by regulating vascular structure (26); Trem2^+^CD9^+^ macrophage populations are related to lipid uptake and are significantly increased in obese populations with increased fat in adipose tissue (27). In T2D-related fatty liver, metabolic Kupffer cell subtypes are related to dietary fat intake and mediate liver inflammation (28). In summary, robust functional studies have shown that monocytes/macrophages are involved in the development of T2D, and our MR analysis further supports the immune pathogenesis of T2D.

T2D is a chronic disease caused by multiple factors. Despite robust studies on the pathogenesis of T2D, the etiology remains unknown. Notably, recent studies have highlighted the significant impact of immunity on T2D incidence, progression, and risk (6), particularly in patients with SIRD and MOD, as evidenced by increased levels of inflammatory markers in their circulation, such as interleukin-6, caspase-8, and S100 calcium-binding protein A12 (EN-RAGE), as well as an increase in WBC count, monocyte count, and CD4^+^ T cell count (10). However, these observational studies only confirmed the involvement of immune cells and inflammation in the development of T2D but cannot provide reliable evidence of causality. Thanks to recent large-scale GWASs on blood traits and T2D, we can use two-sample MR studies to systematically evaluate the causal relationship between peripheral blood immune cells and T2D, with the aim of providing an immune-based perspective for the clinical diagnosis and intervention of T2D. Unfortunately, we can only access GWAS data for T2D compared to healthy individuals, and we cannot find GWAS summary statistics for T2D classified according to SIDD, SIRD, MOD, and MARD subtypes. Further MR analysis of the causal relationship between circulating immune cells and T2D subtypes will provide more evidence-based guidance for clinical practice.

Recent studies have shown that T cells isolated from the peripheral blood and adipose tissue of T2D patients tend to have a pro-inflammatory phenotype, which can promote the development of insulin resistance (29). Compared with healthy individuals, the number of NK cells in the peripheral blood of T2D patients is significantly reduced (30); however, the number of IL-6Rα^+^ NK cells is significantly increased in the peripheral blood of obese patients (31). While CD4 Tregs and B cells are believed to be involved in the pathological process of type 1 diabetes (32, 33), their role in T2D is still under investigation. In this study, we analyzed the causal relationship between lymphocyte subtypes, including CD4^+^CD8^dim^ T cell, CD8^+^ T cell, NKT, CD4 Treg, NK, and B cell count, and T2D based on recently published GWAS data using MR analysis. The results showed that the absolute count of CD4^+^CD8^dim^ and CD8^+^ T cells may contribute to increased T2D risk, which is consistent with previous reports (10, 34). Functional studies have revealed that increased infiltration of both CD4^+^ and CD8^+^ T cells could facilitate insulin resistance development, probably through modulating vascular regeneration (34, 35). However, due to the relatively small sample size of the second GWAS, there are fewer identified instrumental variables that meet the criteria, necessitating further validation with larger-scale GWAS summary statistics.

Overall, our findings suggest that an increased level of circulating monocyte cell count may contribute to T2D development. While circulating lymphocyte cell count was not causally associated with T2D susceptibility, lymphocyte subtypes including CD4^+^CD8^dim^ T cell and CD8^+^ T cell count may predict the risk of T2D. The findings suggest that certain immune cell types and genetic susceptibilities could be used as biomarkers for T2D risk, which could lead to earlier diagnosis and more effective treatment options.

## Data availability statement

The original contributions presented in the study are included in the article/**Supplementary Material**. Further inquiries can be directed to the corresponding author.

## Ethics statement

Ethical review and approval was not required for the study on human participants in accordance with the local legislation and institutional requirements. Written informed consent from the patients/participants or patients/participants’ legal guardian/next of kin was not required to participate in this study in accordance with the national legislation and the institutional requirements.

## Author contributions

JL: study design, data analysis, and manuscript drafting. QN and AW: data analysis and manuscript revision. YZ and LH: manuscript revision. HW: study design and manuscript revision. All authors contributed to the article and approved the submitted version.
