# Supplementary material for: Causal relationship between circulating immune cells and the risk of type 2 diabetes: a Mendelian randomization study
Source: Front Endocrinol (Lausanne). 2023 May 25;14:1210415. doi: 10.3389/fendo.2023.1210415 (PMC10247959; doi:10.3389/fendo.2023.1210415)
Supplement: Supplementary file 1 [file DataSheet_1.docx]

Supplementary Material

Causal Relationship between Circulating Immune Cells and the Risk of Type 2 Diabetes: A Mendelian Randomization Study

Jin Li, Qingmin Niu, Aiwei Wu, Yuchu Zhang, Liquan Hong* and Hu Wang*

***** Corresponding Author: hongliquan@126.com (Liquan Hong); [wanghu19860315@163.com](mailto:wanghu19860315@163.com) (Hu Wang)

SUPPLEMENTARY FIGURE 1

SUPPLEMENTARY FIGURE 2

SUPPLEMENTARY TABLE 1

SUPPLEMENTARY TABLE 2

SUPPLEMENTARY TABLE 3

SUPPLEMENTARY TABLE 4

SUPPLEMENTARY TABLE 5

SUPPLEMENTARY TABLE 6

SUPPLEMENTARY TABLE 7

SUPPLEMENTARY TABLE 8

SUPPLEMENTARY TABLE 9

SUPPLEMENTARY TABLE 10

##
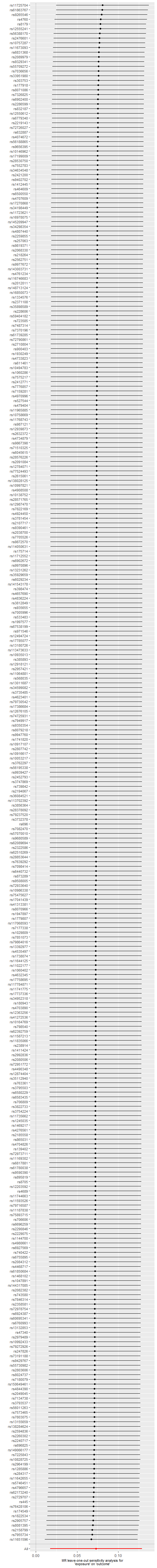
Supplementary Figures

## MR leave-one-out sensitivity analysis for “monocyte cell count” on “T2D”


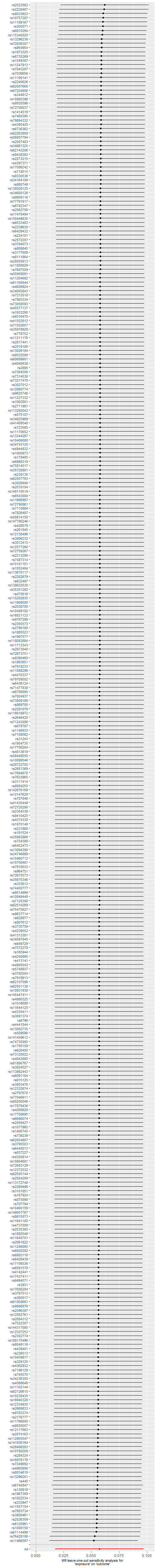


MR leave-one-out sensitivity analysis for “WBC” on “T2D”

**Supplementary Fig.1** **Forest plots for the Mendelian randomization (MR) leave-one-out analysis between circulating immune cells and T2D liability.** Within each panel, the black points represent the causal estimate after discarding each SNP in turn. Red points represent the pooled IVW estimates. Horizontal lines denote 95% confidence intervals.


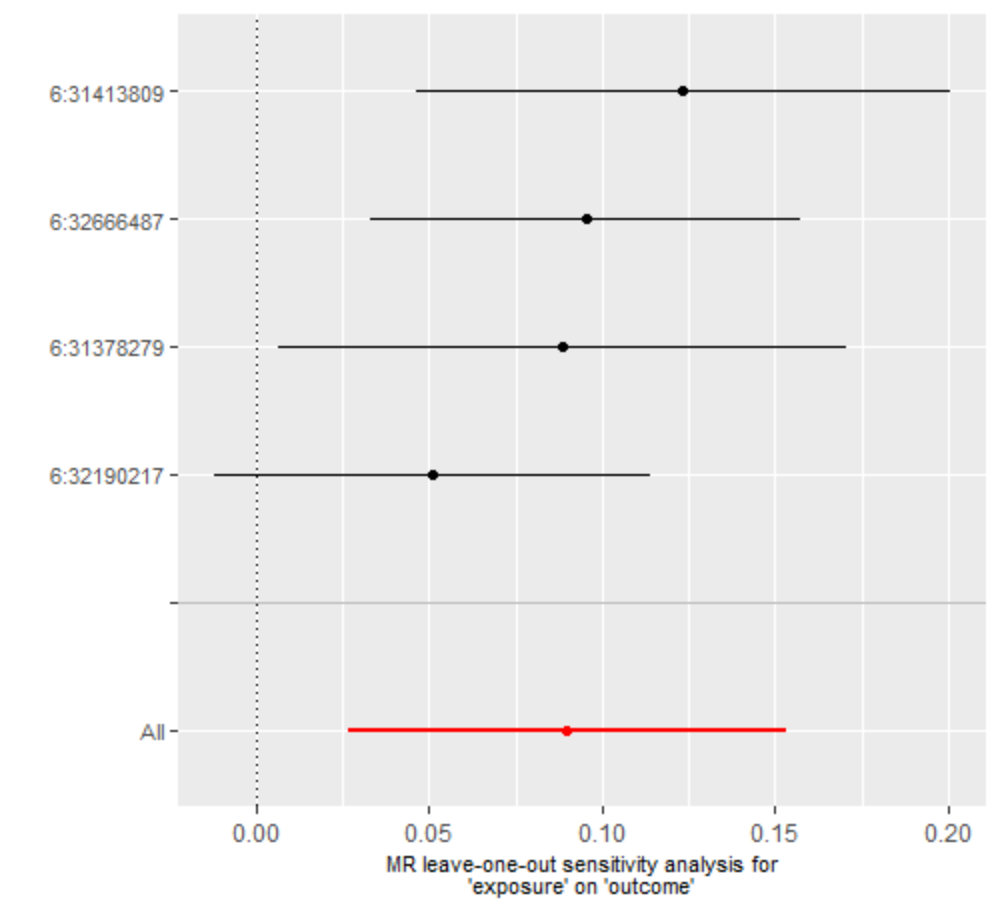


MR leave-one-out sensitivity analysis for “CD8^+^ T cell” on “T2D”


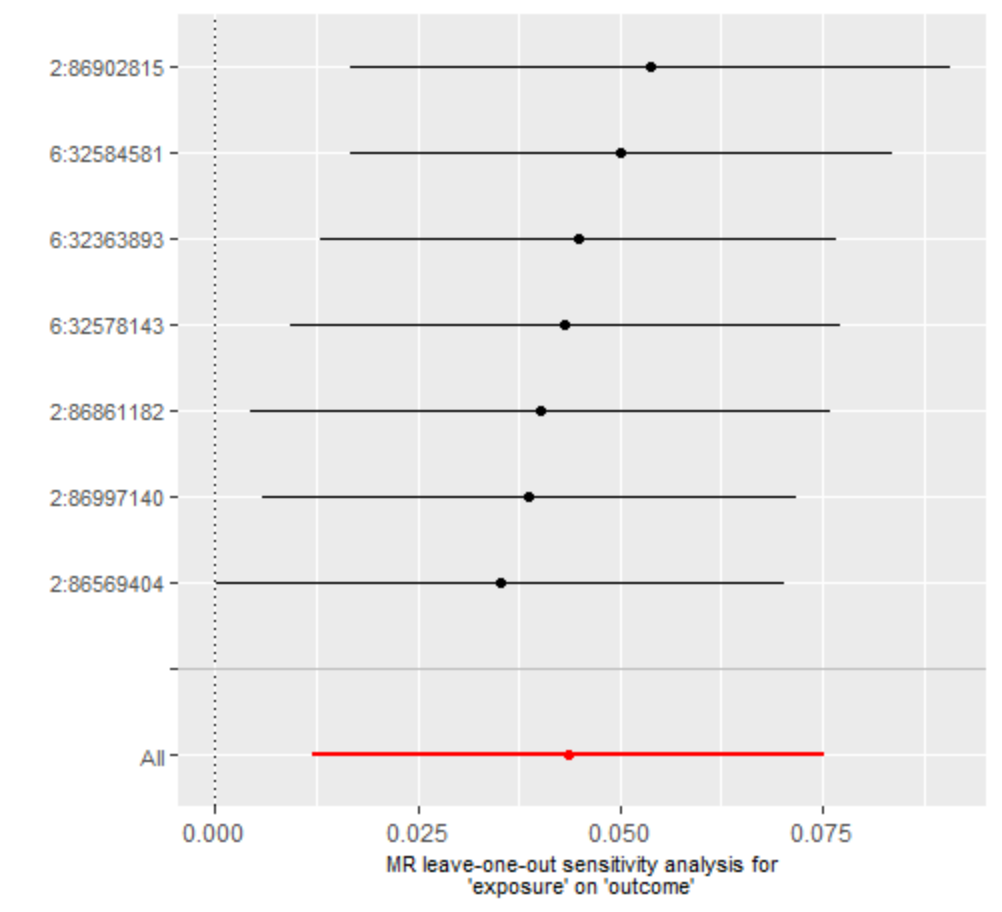


MR leave-one-out sensitivity analysis for “CD4^+^ CD8^dim^ T cell” on “T2D”

**Supplementary Fig. 2** **Forest plots for MR leave-one-out analysis between absolute count of lymphocyte subsets and T2D risk.** Within each panel, the black points represent the causal estimate after discarding each SNP in turn. Red points represent the pooled IVW estimates. Horizontal lines denote 95% confidence intervals.
